# Role of Annexin A2 isoform 2 on the aggregative growth of dermal papillae cells

**DOI:** 10.1042/BSR20180971

**Published:** 2018-12-07

**Authors:** Jing Gu, Yinni Ma, Lijia Yang, Feng Wang, Cao Lei, Jianxin Zhai, Rushan Xia

**Affiliations:** 1 Department of Dermatology, The Affiliated Wuxi No. 2 People’s Hospital of Nanjing Medical University, Wuxi 214002, P.R. China; 2 Department of Ophthalmology, The Affiliated Wuxi No. 2 People’s Hospital of Nanjing Medical University, Wuxi 214002, P.R. China

**Keywords:** Annexin A2, comparative proteomics, dermal papillae cells, overexpression, siRNA

## Abstract

The dermal papilla is a major component of hair, which signals the follicular epithelial cells to prolong the hair growth process. Human Annexin A2 was preliminarily identified by two-dimensional gel electrophoresis (2-DE), MALDI-TOF-MS and database searching. The aim of the present study was to explore the role of Annexin A2 in the aggregative growth of dermal papillae cells (DPC). Reverse transcription-polymerase chain reaction (RT-PCR) and Western blot were adopted to detect the expression of Annexin A2. And siRNA technique was used to suppress the expression of Annexin A2. Construction of over-expression vector was used to up-regulate the expression of Annexin A2. Cell Counting Kit 8 (CCK-8) and proliferating cell nuclear antigen (PCNA) were taken to detect the proliferation of DPC. The expression of Annexin A2 mRNA was up-regulated in passage 3 DPC compared with passage 10 DPC by RT-PCR. In line with the results at the mRNA level, Western blot analysis revealed that Annexin A2 isoform 2 was up-regulated significantly in passage 3 DPC compared with passage 10 DPC. The Annexin A2 isoform 2 siRNA was synthesized and transfected into passage 3 DPC. RT-PCR data showed the mRNA expression of Annexin A2 isoform 2 was suppressed in passage 3 DPC. Western blot results showed the expression level of Annexin A2 isoform 2 and PCNA were suppressed in passage 3 DPC. CCK-8 results showed that the proliferation of passage 3 DPC was suppressed (*P* < 0.05). Recombinant plasmid PLJM-Annexin A2 isoform 2-expression vector were constructed and were transfected into passage 10 DPC. RT-PCR data showed the mRNA expression of Annexin A2 isoform 2 was up-regulated in passage 10 DPC. Western blot results showed the expression level of annexin A2 isoform 2 and PCNA were up-regulated in passage 10 DPC. CCK-8 assay showed the proliferation of DPC was stimulated compared with control group (*P < 0.05). Our study proved that Annexin A2 isoform 2 may participate in regulating the proliferation of DPC and may be related to aggregative growth of dermal papilla cells. Therefore, our study suggests that Annexin A2 may be linked to hair follicle growth cycle.

## Introduction

The dermal papillae cells (DPC) are specialized mesenchymal cells which are believed to induce and regulate hair follicle morphogenesis and growth [1,2]. Aggregative growth is one of the significant properties of the DPC, which is associated with development and cycle regulation of hair follicles [3–6]. However, DPC could lose the aggregative behavior and hair-inducing activity in later passage number [7]. However, up to now, we still know very little about the cellular mechanisms underlying the aggregative behavior of DPC. In order to study the aggregative behavior of DPC, a variety of techniques have been used to identify proteins/genes related to the aggregative property of DPC, including analyzing mRNA expression levels of aggregation related genes [8,9]. Studies have proved that DPC with aggregative behavior could produce soluble molecules and these molecules could help the DPC which lost aggregative behavior regain aggregative behavior [10]. Recent studies indicate that DPC-exosomes contribute to the regulation of hair follicle growth and development [11]. In our previous study, we have got the cytoplasma proteins of DPC and conducted two-dimensional gel electrophoresis (2-DE) analysis. Typical Coomassie brilliant blue-stained gels were shown with several hundred spots clearly identifiable in each gel. We identified 15 proteins, including human Annexin A2 [12].

Annexin A2 is a multifunctional calcium-dependent phospholipid binding protein which has been described as an intracellular protein, cell membrane associated proteins. It involves in endocytic and exocytic events [13]. The overexpression of annexin A2 has been linked to a variety of tumors [14] and keloid [15]. Annexin A2 may participate in keloid formation by inhibiting keloid fibroblast proliferation and may be a valuable therapeutic target for keloid lesions [16]. But the role of Annexin A2 in hair follicle and dermal papillae is not reported. In order to examine the difference of Annexin A2 expression level in passages 3 and 10 DPC, we adopted reverse transcription-polymerase chain reaction (RT-PCR) and Western blot. We found that annexin A2 was up-regulated significantly in passage 3 DPC compared with that in passage 10 DPC. Additionally, We transfected Annexin A2 siRNA and PLJM-Annexin A2 expression vector to down-regulate and up-regulate the expression of Annexin A2 in DPC. Cell Counting Kit 8 (CCK-8) and PCNA were used to investigate the role of Annexin A2 on the proliferation of DPC.

## Materials and methods

### Dermal papilla separation and cultures

The study using clinical samples was approved by the Review Board of the Affiliated Wuxi No. 2 People’s Hospital of Nanjing Medical University. The human non-balding scalp-skin samples (*n* = 2) were from Affiliated Wuxi No. 2 People’s Hospital of Nanjing Medical University with the informed consent of donors and approval of Wuxi second hospital ethical committee (20161209). Experiments conformed to the principles set out in the WMA Declaration of Helsinki and the Department of Health and Human Services Belmont Report.

We used improved two-step enzyme method set up previously in our laboratory to isolate the dermal papillae [17]. Firstly, the skin was sterilized and digested in 0.5% (w/v) dispase (Sigma, U.S.A.) for 12–16 h at 4°C and in 0.2% (w/v) collagenase D (Sigma, U.S.A.) for 6 h at 37°C sequentially. The digested tissue was then centrifuged at 550–850 ***g*** for 3–5 min. The dermal papillae deposited at the bottom of tube as a clump of cells, whereas other cells floated in the supernatant. After centrifuged for several times, the human dermal papillae were separated from other types of cells. The human dermal papillae were cultured in DMEM medium (Gibco, U.S.A.) containing 10% fetal calf serum (Gibco, U.S.A.) and incubated at 37°C in an atmosphere of 95% air and 5% CO_2_.

### Different expression of Annexin A2 mRNA between passage 3 and passage 10 DPC

Total RNA was extracted from passage 3 and passage 10 DPC by using Trizol (Invitrogen, U.S.A.) according to the manufacturer’s instruction. c-DNA was synthesized from 1 µg total RNA using OligodT primers by reverse transcription reagent (Takara, Japan) based on the instruction of the manufacturer. DNA were amplified using Taq DNA Polymerase (Takara, Japan) with the following primers: β-actin (618 bp), 5′-CGG GAC CTG ACT GAC TAC CTC-3′ and 5′-CAA GAA AGG GTG TAA CGC AAC-3′; Annexin A2 (329 bp), 5′-TGAAGTCAGCCTTATCT GGC-3′ and 5′-ATTGACCAAGATGCTCGG-3′. Amplified fragments were separated through electrophoresis on 1% agarose gels and visualized by ethidium bromide staining. The experiment was repeated three times.

### Different expression of Annexin A2 between passage 3 and passage 10 DPC

The expression of Annexin A2 in DPC at passage 3 and passage 10 was measured through the Western blot assay. After cell lysis and centrifugation (14000 rpm × 15 min), protein was subjected to a SDS-PAGE gel and then, transferred to polyvinylidene fluoride (PVDF) membrane. After blocking with TBST containing 5% skim milk, the membranes were first incubated with mouse anti-human Annexin A2 mAb (diluted 1:1000, Proteintech Group, China) or anti-PCNA Mouse mAb (dilution 1:1000, Affinity Group, U.S.A.) at 4°C overnight, and then with peroxidase-conjugated goat anti-mouse immunoglobulin (Beyotime, China) diluted 1:1000 in Tris-Buffered Saline Tween-20 (TBST) for 1 h. Finally, PVDF membranes were washed three times with TBST, and protein bands were visualized with ECL Substrates (Thermo, U.S.A.). The experiment was repeated three times.

### Design and synthesis of siRNA

The siRNA oligonucleotide targeting Annexin A2 isoform 2 was designed and synthesized by Gene Pharma (Shanghai, China) based on the published sequence of Annxin A2 isoform 2 (sense 5′-GCAUAGCAACUUCGGAUUUTT-3′; antisense 5′-AAAUCCGAAGUUGCUAUGCTT-3′). Meanwhile, siRNA for negative control (sense 5′-UUCUCCGAACGUGUCACGUTT-3′; antisense 5′-ACGUGACACGUUCGGAGAATT-3′) was synthesized.

### Construction of recombinant plasmid PLJM-Annexin A2 isoform 2

Total RNA extracted from human DPC. cDNA was synthesized from mRNA by reverse transcription reagent. The full length cDNA encoding Annexin A2 isoform 2 was obtained through polymerase chain reaction (Promega, U.S.A.). The primers used for PCR amplification were: F 5-AATAATACCGGTAGTTCTACTGTTCACGAAATC-3 and R 5-AATAATTTCGAATCAGTCATCTCCACCACAC-3. PCR-amplified DNA fragments were separated by electrophoresis on 1% agarose. The PCR products were visualized on agarose gel by staining with ethidium bromide. cDNA of Annexin A2 was obtained from the gel used agarose gel DNA extraction kit (Takara, Japan), then cloned into PLJM (the expression plasmid) and placed between Age I and Bstb I restriction sites. The insert was sequenced and validated to be in complete agreement with the expected sequence.

### Down-regulate the expression of Annexin A2 isoform 2 in DPC

Down-regulating the expression of Annexin A2 was performed by using Annexin A2 isoform 2 siRNA. Passage 3 DPC were diluted in fresh media without antibiotics 24 h before transfection and later transferred to six-well plates. When the DPC had grown to a confluence of 70–80%, they were transfected with 100 pM of siRNA and 5 ul of lipo2000 (Invitrogen, U.S.A.) per well. The medium was changed 6 h after the transfection. The experiment was repeated three times.

### Up-regulate the expression of Annexin A2 isoform 2 in DPC

We transfected passage 10 DPC with PLJM-Annexin A2 isoform 2 to up-regulate expression of Annexin A2 isoform 2. Passage 10 DPC were transferred to six-well plates 24 h before transfection. When the DPC had grown to a confluence of 70–80%, they were transfected with 4 ug of PLJM-Annexin A2 isoform 2 and 10 ul of lipo2000 per well. The medium was changed 6 h after the transfection. The experiment was repeated for three times.

### Detect the efficiency of transfection

Real-time fluorescent quantitative PCR and Western Blot were applied to examine transfection efficiency. After 48 h of transfection, total RNA of transfected cells were extracted by Trizol. cDNA was synthetized by reverse transcription reagent (Takara, Japan). Real-time PCR was carried out by using SYBR green PCR master mix (Qiagen, Germany). Primers for fluorescent quantitative real-time PCR consisted of β-actin (F-5′-ACTGGAACGGTGAAGGTGAC-3′ and R-5′-AGAGAAGTGGGGTGGCTTTT-3′) and Annexin A2 (F-5′- ATTGCCTTCGCCTACC-3′ and R-5′-GCTCTTCTACCCTTTGC-3′). 48 h after the transfection, cells were lysed in Radio Immuno Precipitation Assay buffer with protease inhibitor. Protein samples were loaded in 10% SDS-PAGE gel, then transferred onto PVDF membrane and blotted by anti-Annexin A2 antibody followed by the HRP-conjugated secondary antibody. Signals were visualized using ECL Substrates. β-actin served as the control group. The experiment was repeated three times.

### Cell proliferation assay

#### CCK-8 cell proliferation assay

Cell proliferation rates were measured by using CCK-8 (Dojindo, Japan). Briefly, 0.5 × 104 transfected cells/well were seeded in a 96-well plate and further incubated for 2, 12, 24, 48, and 72 h, respectively. CCK-8 reagent (10 ul) was added to each well and incubated for 2 h. Then, OD450 nm value in each well was verified by a microplate reader. The experiment was repeated three times.

#### Proliferating cell nuclear antigen (PCNA)

The expression of proliferating cell nuclear antigen (PCNA) in DPC was measured through the Western blot assay. The method is the same as section ‘Different expression of Annexin A2 between passage 3 and passage 10 DPC’.

## Results

### Identification of Annexin A2 by MALDI-TOF MS

**Figure 1:**
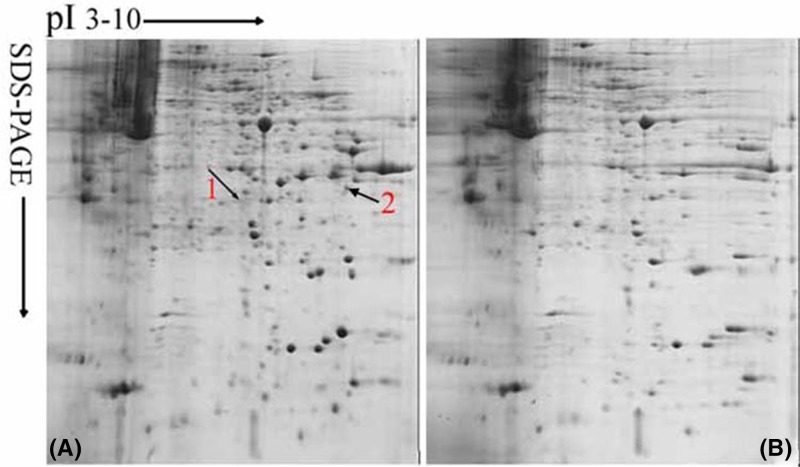
2-DE patterns of cytoplasma proteins in passage 3 DPC(A) and passage 10 DPC(B) 2-DE patterns of cytoplasmic protein in passage 3 DPC (**A**) and passage 10 DPC (**B**). Protein (1.0 mg) was loaded and separated in IPG strips (pI range of 3–10), and the gels were stained with Brilliant Blue G-250. They yielded good spectra (Figure 2A,B).

To study the proteins involved in aggregation of DPC, cytoplasma proteins were subjected to 2-DE analysis. Typical Coomassie brilliant blue-stained gels were shown in Figure 1. Several hundred spots were clearly identifiable in each gel. Human Annexin A2 (spot 1) and Annexin A2 isoform 1 (spot 2) proteins were identified. We selected them for further study.

**Figure 2:**
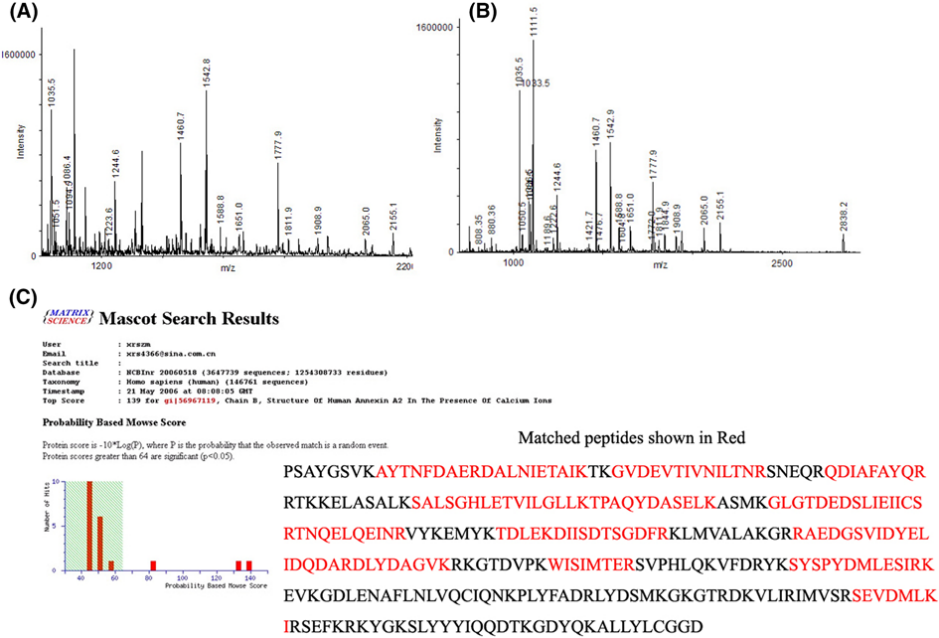
Typical MALDI-TOF-MS spectrum of spot 1 from the 2-DE map The MS spectrum of the peptide mixture was obtained from a typical in-gel digestion of the 2-DE separated protein spot 1 (**A**) and spot 2 (**B**). (**C**) Mascot search results of spot 1 and 2 which are matched with Annexin A2 isoform 1 and Annexin A2 isoform 2. Peptides from spot 1 are shown in bold red. Number of mass values searched is 55 and number of mass values matched is 18. Sequence coverage is 51%.

### Expression of Annexin A2 in DPC at passage 3 and passage 10

**Figure 3:**
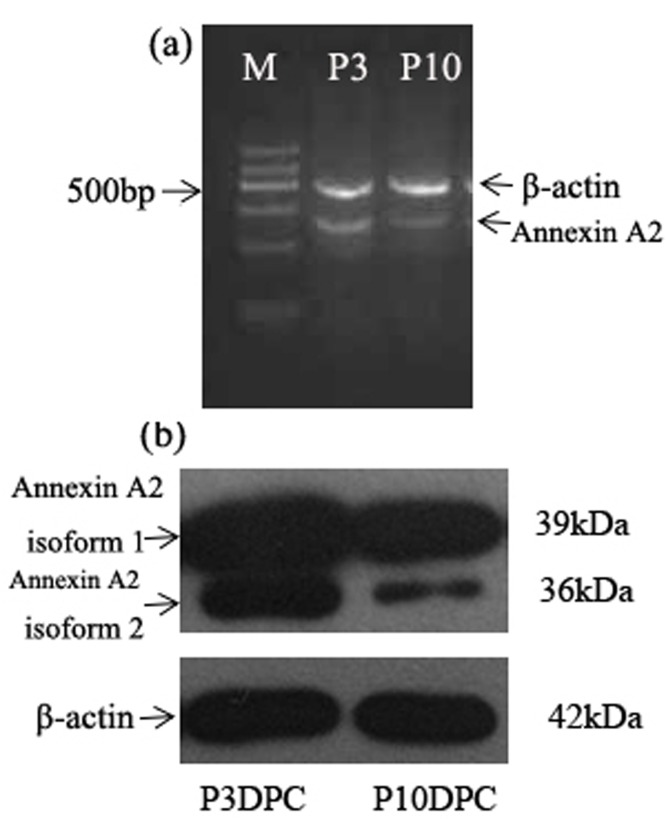
Expression of Annexin A2 in mRNA and protein levels (**A**) Comparative expression mRNA of Annexin A2 in passage 3 and passage 10 DPC. In line with the results at the 2-DE level, the RT-PCR results revealed down-regulation of Annexin A2 in passage 10 DPC. (**B**) Western blot analysis revealed that Annexin A2 isoform 2 was up-regulated in passage 3 DPC and down-regulated in passage 10 DPC.

RT-PCR and Western blot were adopted to detect the expression level of Annexin A2 in passage 3 and passage 10 DPC as well. The expression of mRNA of Annexin A2 in passage 3 and passage 10 DPC was shown (Figure 3A). The expression of Annexin A2 in passage 3 DPC was compared with that in passage 10 DPC through Western blot analysis (Figure 3B).

### Effect of inhibiting the expression of Annexin A2 isoform 2 by siRNA on biological function of passage 3 DPC

**Figure 4:**
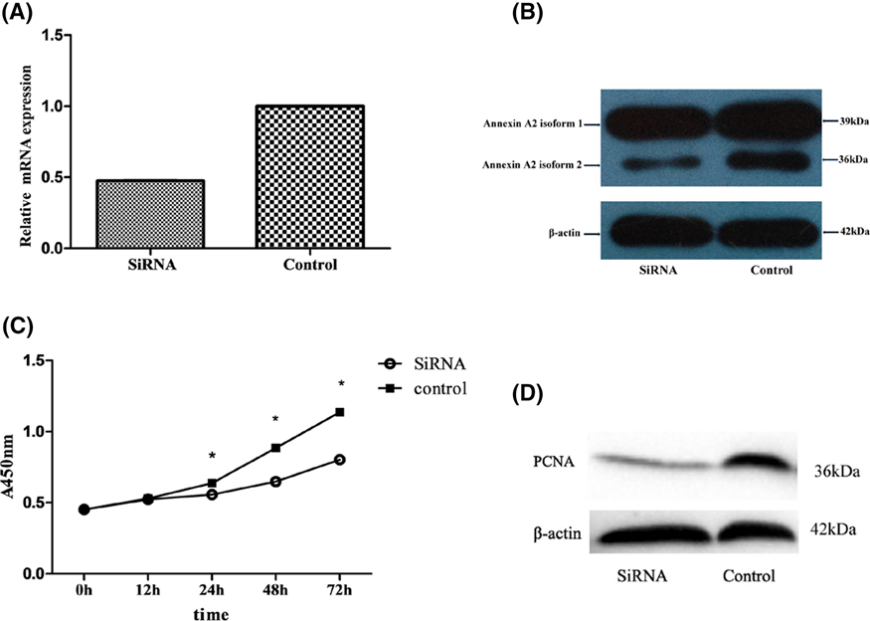
Effect of RNA interference mediated inhibition of Annexin A2 isoform 2 expression on biological function of passage 3 DPC (**A**) Real-time fluorescent quantitative PCR data showed the mRNA expression of Annexin A2 was suppressed in passage 3 DPC with aggregation (*P* < 0.05). (**B**) Western blot results showed the expression level of Annexin A2 isoform 2 was suppressed in DPC. (**C**) CCK-8 assay showed that the proliferation of DPC which transfected with Annexin A2 isoform 2 siRNA was suppressed (*P < 0.05). (**D**) Western blot results showed the expression level of PCNA was suppressed in passage 3 DPC.

In order to clarify whether the proliferation of DPC is influenced in response to down-regulating Annexin A2 isoform 2, we adopted siRNA technology to suppress the expression of Annexin A2 isoform 2 in passage 3 DPC. When compared with the control group, the expression of Annexin A2 was obviously decreased in DPC transfected with Annexin A2 isoform 2 siRNA (Figure 4A,B). CCK-8 and PDCA assay demonstrated that the proliferation of DPC were significantly decreased following transfection of Annexin A2 isoform 2 siRNA (Figure 4C,D).

### Effect of up-regulating expression of Annexin A2 isoform 2 on biological function of 10 passage DPC

**Figure 5:**
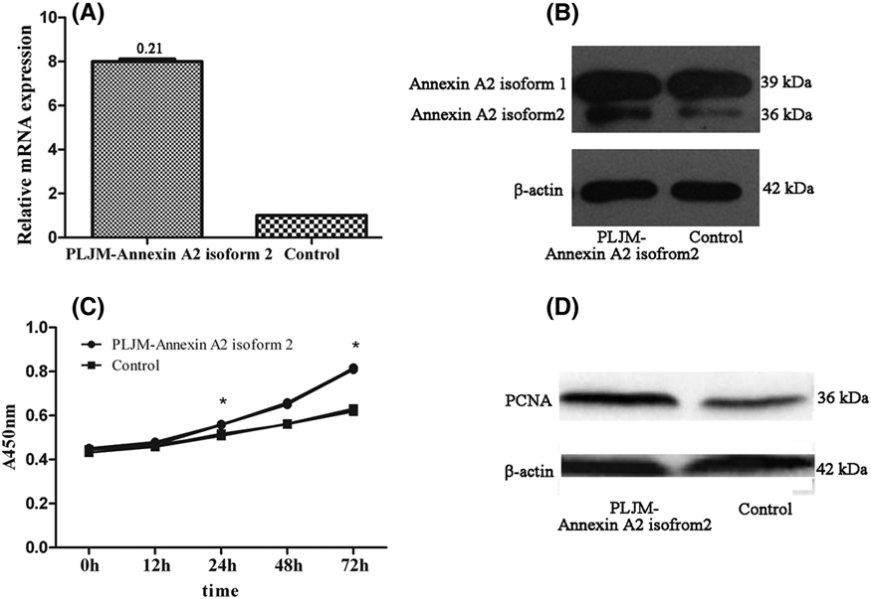
Effect of up-regulating expression of Annexin A2 isoform 2 on biological function of passage 10 DPC (**A**) Real-time fluorescent quantitative PCR data showed the mRNA expression of Annexin A2 was up-regulated in passage 10 DPC (*P* < 0.05). (**B**) Western blot results showed the expression level of Annexin A2 isoform 2 was up-regulated in passage 10 DPC. (**C**) CCK-8 assay showed the proliferation of DPC which transfected with PLJM-Annexin A2 isoform 2 was stimulated compared with control group (*P < 0.05). (**D**) Western blot results showed the expression level of PCNA was up-regulated in passage 10 DPC.

As we confirmed that the expression of Annexin A2 isoform 2 was different in passage 3 and passage 10 DPC, we subsequently tried to identify the regulatory effect of Annexin A2 isoform 2 on the biological function of DPC. We applied lipo2000 to transfect the expression vector of PLJM-Annexin A2 isoform 2 to DPC to achieve increased expression. Real-time fluorescent quantitative PCR detection showed stronger expression of Annxin A2 mRNA in the PLJM-Annexin A2 isoform 2 transfection group than that in the control group (Figure 5A). Compared with the control group, the expression of Annexin A2 isoform 2 was notably stronger in the PLJM-Annexin A2 isoform 2 transfection group (Figure 5B). CCK-8 and PCNA assays indicated that the proliferation of DPC was significantly enhanced following transfection of PLJM-Annexin A2 isoform 2 (Figure 5C,D).

## Discussion

Proteomics permits the large-scale and high-throughput analysis of proteins and has become a powerful tool with which to study the total or specific proteins from cells. Aggregative growth is one of the significant properties of the DPC. The ability of new hair regeneration *in vivo* is associated with aggregative behavior *in vitro* [18,19]. In culture, dermal papilla cells have maintained their hair inductive properties before passage four [20] and become growth-exhausted after five-ten sub-passages [21]. However, we still know very little about the cellular mechanisms underlying the aggregative behavior of DPC. In the previous study, we analyzed proteins expressed in DPC with the characters of aggregation by proteomic strategy. Annexin A2 was preliminarily identified.

Annexin A2 is a widely distributed, highly conserved, peripheral membrane protein expressed abundantly on endothelial cells, macrophages, myeloid cells and some tumor cells [22]. It belongs to a family of proteins characterized by their Ca^2+^-dependent binding to negatively charged phospholipids. It binds actin, mRNA, tissue plasminogen activator (tPA), plasminogen, and so on [23–26]. Reflecting its multiple functions, Annexin A2 is found at various cellular locations. It resides soluble in the cytoplasm or is associated with the actin cytoskeleton. Annexin A2 is related to intracellular membrane compartments of both the endo- and exocytic pathways [27].

Annexin A2 plays a vital role in the regulation of cellular growth and in signal transduction pathways. As a tumor-associated protein, Annexin A2 promotes cancer progression including proliferation, invasion and metastasis in nasopharyngeal carcinoma, ovarian cancer, gliomas, hepatomas, pancreatic cancer and so on [28–31]. As an autocrine/paracrine factor, Annexin A2 enhances osteoclast formation and bone resorption [32]. As an inhibitor, Annexin A2 participates in human skin keloid formation [16]. All of these indicate that Annexin A2 may be a multifunctional protein in different cells.

In the present study, we adopted RT-PCR and Western blot to probe the expression level of Annexin A2 in different DPC with and without aggregation. RT-PCR results revealed the down-regulation of Annexin A2 in passage 10 DPC (without aggregation). Western blot results demonstrate annexin A2 was up-regulated significantly in passage 3 DPC (with aggregation) compared with passage 10 DPC (without aggregation). In order to examine the role of Annexin A2 on the proliferation of DPC, we employed RNA interference and PLJM-Annexin A2 expression vector transfection to down-regulate and up-regulate the Annexin A2 expression in DPC. CCK-8 was applied to detect the proliferation of DPC. The results illustrated that down-regulation of Annexin A2 resulted in decreased proliferation of passage 3 DPC (with aggregation), and up-regulation of Annexin A2 resulted in increased proliferation of passage10 DPC (without aggregation). All of these indicate that Annexin A2 may participate in regulating the proliferation of DPC. Wnt/β-catenin signaling pathway is sufficient to maintain anagen-phase characteristics of dermal papilla cells [33]. As a key factor of Wnt signaling pathway, TCF4 is closely related with DPC proliferation and its biological function [34]. Whether Annexin A2 plays a role through the Wnt/β-catenin signaling pathway remains to be confirmed by further studies.

Our study may provide a vision that Annexin A2 is related to the aggregative behavior of dermal papilla cells and may be a marker of DPC with aggregation. Nevertheless, whether it is involved in the hair follicle growth cycle directly or through other ways still need to be further investigated.

## Supporting information

## Abbreviations

2-DE: two-dimensional gel electrophoresis
CCK-8: Cell Counting Kit 8
DPC: dermal papillae cells
PCNA: proliferating cell nuclear antigen
PVDF: polyvinylidene fluoride
