# Supplementary material for: Role of Annexin A2 isoform 2 on the aggregative growth of dermal papillae cells
Source: Biosci Rep. 2018 Dec 7;38(6):BSR20180971. doi: 10.1042/BSR20180971 (PMC6435533; doi:10.1042/BSR20180971)
Supplement: Supplementary file 1 [file bsr20180971_Supp1.pdf]

无锡市第二人民医院科研伦理审查申请表

一、科研项目名称：

Annexin A2 在人毛囊毛乳头细胞凝集生长中的作用研究

二、类别：基础性研究 ☑　　应用性研究☐　　发展性研究☐　　护理研究☐

其他（请注明）

三、研究或者相关技术应用方案的简要说明（100 字以内）：

本项目拟取健康人头皮毛囊，分离并培养毛乳头，通过 RNAi 技术和基因过表达技术研究 Annexin A2 在毛乳头细胞凝集和非凝集生长状态下的表达，并观察毛乳头细胞的生长和增殖情况，明确 Annexin A2 在毛乳头细胞凝集生长中的作用，为后续的分子机制研究奠定基础。

四、承担科室：皮肤科　　项目负责人：夏汝山

五、参加课题研究相关人员资质情况介绍（可附后）

科室	姓名	性别	年龄	职称	承担任务	备注
皮肤科	夏汝山	男	46	副主任医师	课题设计及指导	
皮肤科	杨莉佳	女	58	主任医师	课题指导及实施	
皮肤科	顾静	女	26	研究生	课题实施	
皮肤科	马印尼	女	22	研究生	课题实施	
眼科	王峰	男	27	研究生	数据分析	
皮肤科	曹蕾	女	35	主治医师	标本制备及数据分析	
皮肤科	翟建新	男	55	主任医师	标本制备及数据分析	

六、上交材料清单（勾选）

序号	材料
1☑	新技术、新项目基本情况说明
2☑	无锡市第二人民医院学术委员会新技术、新项目审核意见
3☑	患者知情同意书
4☑	应急预案
5☑	操作者资质证明材料
6☑	大致费用情况
7☑	相关依据资料
8☑	其他材料

申请人责任声明	我将遵循依据《中华人民共和国执业医师法》、《医疗机构管理条例》《涉及人的生物医学研究伦理审查办法（试行）》以及伦理委员会的要求，开展本项实验研究。		
申请人签字	夏汝山	日期	2016.12.06

伦　理　委　员　会　审　查　受　理／

补　充　文　件　通　知　书

伦理委员会审查受理/补充文件通知书

[illegible]的科研伦理审查资料提交/需补充______，同意受理。

受理日期：2016年12月9日，受理编号20161209，

自受理日期起一个星期内拨打电话：0510-66681050 或手机：13951508622 咨询伦理审查具体事宜。备注：联系人：陆胜。联系地址:无锡市中山路 68 号 无锡市第二人民医院医务处。邮编：214002

无锡市第二人民医院

伦理委员会
